# Integrating motion capture technology and intraoral ultrasonography for 3D anatomical analysis

**DOI:** 10.1038/s41598-025-05763-x

**Published:** 2025-07-02

**Authors:** Soo-Bin Kim, Kang-Woo Lee, Hyungkyu Bae, Kyu-Ho Yi, Kyung-Seok Hu, Hee-Jin Kim

**Affiliations:** 1 Department of Oral Anatomy, Institute of Biomaterial Implant, College of Dentistry, Wonkwang University, Iksan, 54538 South Korea; 2 School of Mechanical Engineering, Gwangju Institute of Science and Technology, Gwangju, Republic of Korea; 3 Department of Anatomy, Yonsei University Wonju College of Medicine, 20, Ilsan-ro, Wonju-si, 26462 Gangwon-do South Korea; 4 You and I clinic, Seoul, Korea; 5 Division in Anatomy and Developmental Biology, Department of Oral Biology, BK21 FOUR Project, Human Identification Research Institute, Yonsei University College of Dentistry, 50-1 Yonsei-ro, Seodaemun-gu, Seoul, 03722 Republic of Korea; 6 Department of Electric & Electronical Engineering, College of Engineering, Yonsei University Seoul, Seoul, South Korea

**Keywords:** Motion capture, 3D reconstruction, Ultrasonography, Anatomical analysis, Anatomy, Engineering

## Abstract

The inherent two-dimensional (2D) nature of ultrasonography (US) limits its accuracy in visualizing complex intraoral structures. This proof-of-concept feasibility study introduces a novel approach integrating motion-capture technology and three-dimensional (3D) reconstruction to overcome these limitations and improve anatomical interpretation. Two healthy Korean volunteers underwent real-time 2D B-mode intraoral US (11.5 MHz) targeting the maxillary second molars in the occlusal plane. Magnetic resonance imaging (MRI) and 3D facial scans were performed with a 2.7-cm-wide mouth opening, and the images were superimposed. A motion-capture camera tracked the US probe to verify the 3D positioning of the US images. The integrated system enabled visualization of complex anatomical structures surrounding the coronoid process with greater spatial context. Comparative analysis between US images, 3D models, and MRI confirmed the feasibility of accurately locating intraoral anatomical features with high reproducibility. This feasibility study demonstrates the utility of motion capture and 3D imaging in enhancing intraoral US. Accurate spatial localization is essential due to the dynamic positioning of adjacent structures affecting the temporomandibular joint. The findings provide foundational data for standardizing future intraoral US methodologies and suggest directions for real-time anatomical analysis and procedural guidance, such as US-guided botulinum toxin injection into the temporalis tendon or preoperative implant site planning.

**Supplementary Information:**

The online version contains supplementary material available at 10.1038/s41598-025-05763-x.

## Introduction

Diagnostic imaging of the facial region encompasses panoramic radiography, computed tomography (CT), magnetic resonance imaging (MRI), and ultrasonography (US). Among these, US images not only visualize internal structures in real time but are also noninvasive and radiation-free, making them safe, inexpensive, and convenient; therefore, they are widely used for treatment and diagnosis in the facial area^1,2^. The application scope has expanded from the facial region to the intraoral area. Recent advancements include using intraoral US images to visualize intricate structures such as the temporalis tendon and performing US-guided injections, showcasing the versatility and growing utility of US technology^3–5^.

Despite these benefits, the inherently two-dimensional (2D) nature of US restricts its ability to accurately capture complex spatial relationships between anatomical structures. Visualization of bony landmarks and deep regions remains particularly challenging. In addition, image interpretation is highly operator-dependent, requiring substantial expertise and subject to variability. Artifacts can also arise due to suboptimal probe control, differences in scanning techniques, and tissue–ultrasound wave interactions, further limiting diagnostic reliability^6,7^.

To overcome these limitations, recent medical research has proposed the integration of motion-capture and volume-rendering technologies to enhance the anatomical interpretation of US data. Motion-capture systems enable real-time probe localization relative to anatomical landmarks by tracking the spatial movement of the US probe through an optical tracking system. This reduces variability introduced by manual scanning and improves standardization. Simultaneously, volume-rendering software such as 3D Slicer allows 2D US slices to be stacked into three-dimensional (3D) volumetric representations, offering improved anatomical clarity. This integration improves spatial precision, enables real-time anatomical alignment, and addresses the limitations of conventional 2D US imaging^7,8^.

Therefore, this study aims to validate the feasibility of combining motion-capture technology and 3D reconstruction with intraoral US to enhance spatial accuracy and anatomical identification. Our objective is to provide foundational data for advancing and standardizing intraoral US methodologies, particularly in anatomical regions that are difficult to interpret using traditional techniques.

## Methods

### Subjects

Two healthy Korean volunteers (one female, 34 years old; one male, 58 years old) participated in the study. Both participants underwent all imaging procedures, including MRI, 3D scanning, and ultrasonography. Individuals lacking maxillary second molars, those who had undergone temporomandibular joint therapy, or those diagnosed with abnormalities were excluded. The Yonsei University College of Dentistry Institutional Review Board approved all study procedures (IRB identification code: No. 2–2023-0050; approval date: October 19, 2023). Comprehensive written informed consent, including permission for online open-access publication of potentially identifying images and data, was obtained from all participants (or their legal guardians) prior to enrollment. All methods were performed in accordance with relevant guidelines and regulations and with the principles of the Declaration of Helsinki; participants received a full explanation of the study goals and procedures and were free to withdraw at any time.

### Reference point

**Fig. 1:**
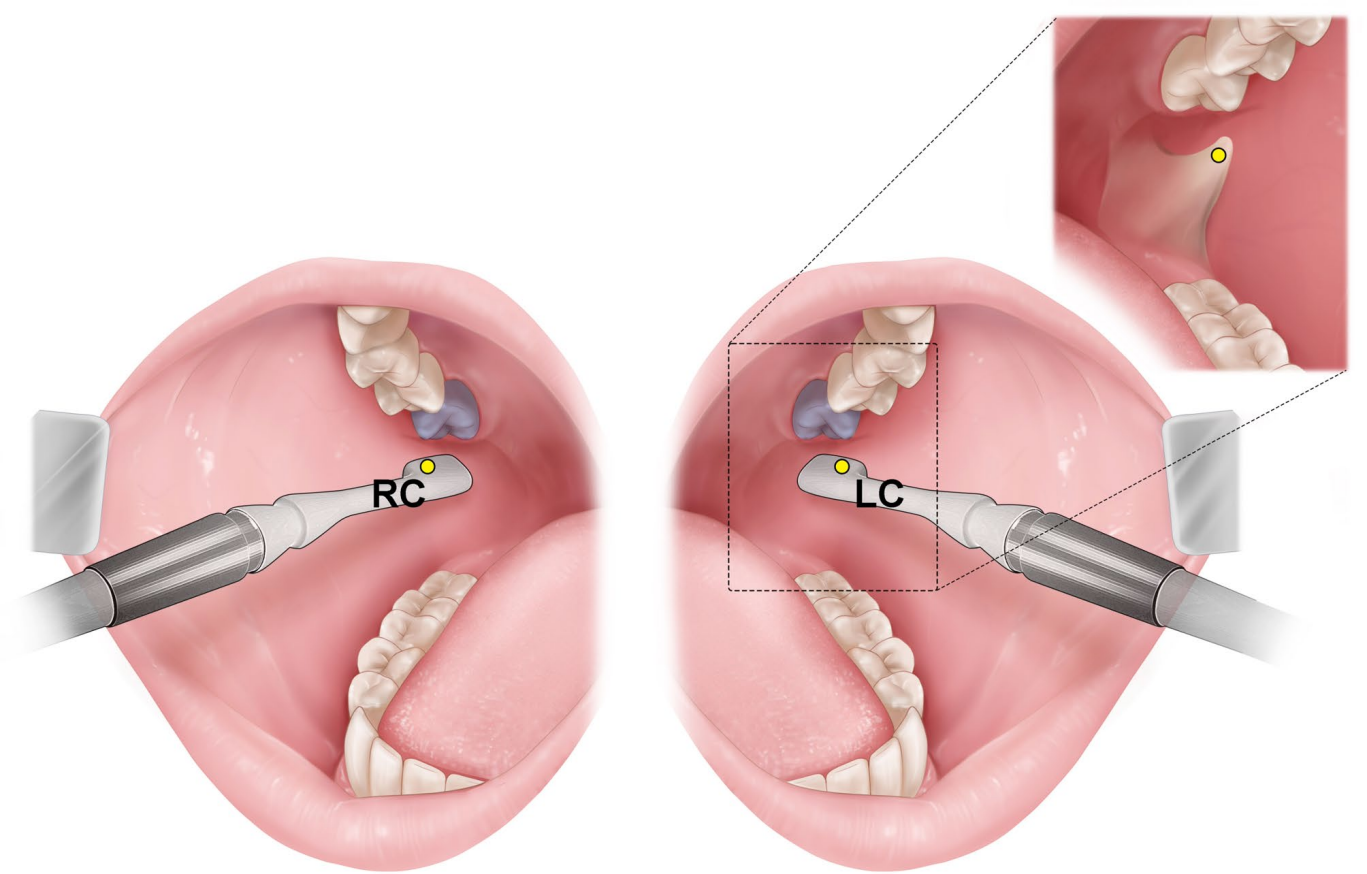
Reference point for the ultrasonography (US) scanning: LC, left cheek mucosa at the second molar (purple) on the occlusal plane; RC, right cheek mucosa at the second molar (purple) on the occlusal plane.

During US examination, all volunteers were positioned in a semi-supine position with their mandibular border aligned parallel to the ground and instructed to maintain a fixed gaze to minimize head movement. Participants’ mouths were opened to a width of 2.7 cm using a mouth prop. Real-time 2D B-mode US images were obtained using a high-frequency (11.5 MHz) intraoral transducer (E-CUBE 15 Platinum; ALPINION Medical Systems). Reference points for intraoral US scanning were the left and right cheek mucosa at the maxillary second molar on the occlusal plane, designated as LC and RC points, respectively (Fig. 1). Bilateral intraoral US images were captured horizontally at the LC and RC points, positioning the transducer just below the occlusal surface of the maxillary second molar. The examiner’s arm was stabilized during scanning to further minimize probe movement and reduce imaging artifacts caused by muscle contractions or soft tissue displacement. The transducer was encased in US gel to ensure precise application and prevent gel from entering the oral cavity.

### MRI scanning

MRI was performed on the volunteer using a 3.0 T scanner (Pioneer, GE Healthcare, Waukesha, WI, USA) equipped with a 16-channel flex large coil, following a mouth opening of 2.7 cm. This study utilized IDEAL-IQ (Iterative Decomposition of water and fat with Echo Asymmetry and Least-squares estimation - Iron Quantification) and T2-weighted axial imaging. The imaging parameters for the T2-weighted image were as follows: echo time, 85 ms; echo train length, 9; repetition time, 3100 ms; bandwidth, 83.33 kHz; NEX (number of excitations), 1.0; field of view, 230 × 230 mm; slice thickness, 4.0 mm; scan time, 2:17 min; flip angle, 111; and matrix, 380 × 320.

### Implementation of 3D superimposed images

**Fig. 2:**
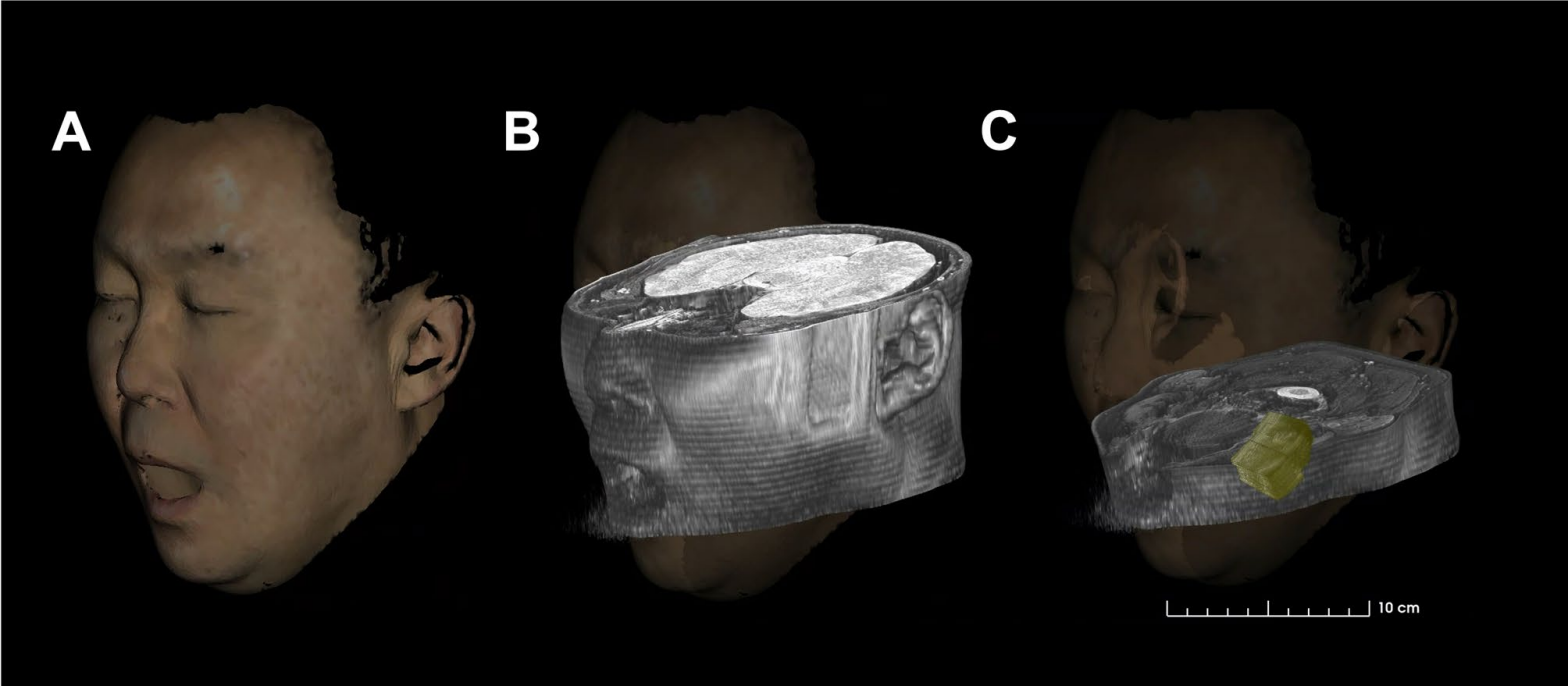
Superimposition process of the three images. Three-dimensional (3D) scanned face (**a**), combined 3D face scan and magnetic resonance imaging with aligned nose and ear (**b**), aligned image with tracked intraoral ultrasonography images at the reference point level (**c**).

**Fig. 3:**
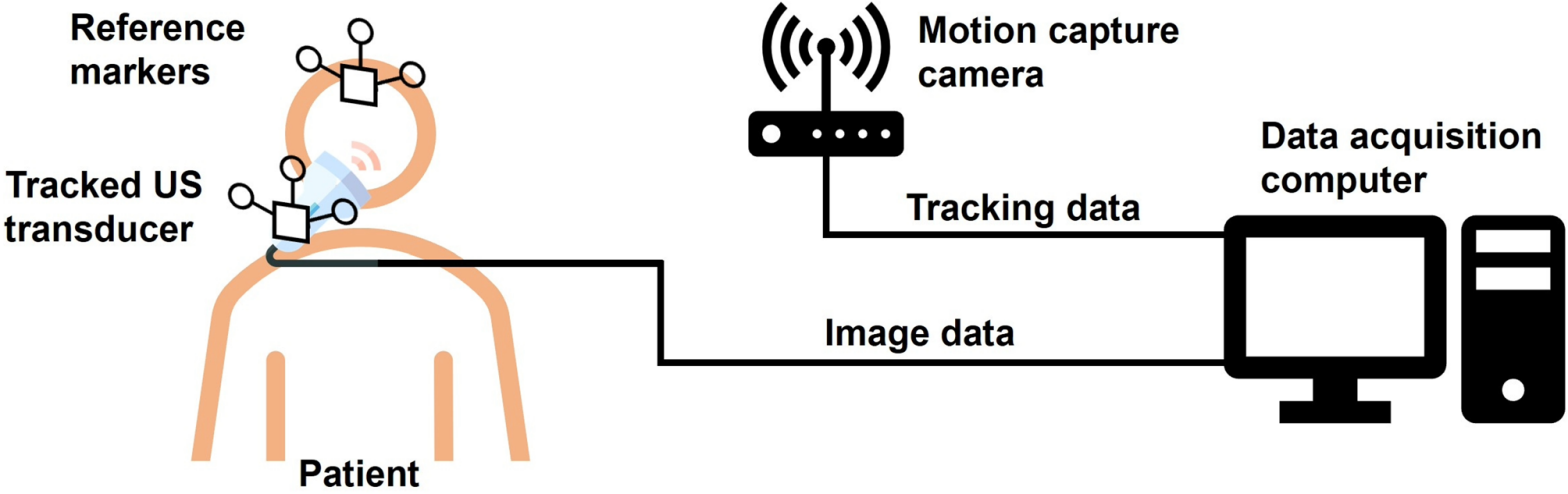
Schematic diagram illustrating the motion-capture–assisted tracking system for intraoral ultrasonography (US). The US transducer and reference markers were tracked in real time using a motion capture camera (OptiTrack V120:Trio), and the patient’s forehead marker defined the reference coordinate system, enabling three-dimensional localization of the US probe relative to anatomical structures.

A surface model of the volunteer’s face was created by scanning with the mouth open to 2.7 cm using an Artec Space Spider 3D scanner (Artec 3D, Luxembourg). This high-resolution scanner utilizes blue light technology, achieving a 0.1 mm 3D resolution and 0.05 mm point accuracy (Fig. 2a). The obtained 3D surface model was superimposed onto MRI and aligned with the nose and ear via 3D Slicer software and its SlicerIGT extension (Fig. 2b). Subsequently, the MRI were aligned to the reference point level. To verify the 3D positional information of the US images, the intraoral probe with an attached marker was tracked using a motion-capture camera (OptiTrack V120: Trio, NaturalPoint, Corvallis, OR, USA). Simultaneously, a marker attached to the participant’s forehead served as the Reference Coordinate System (Fig. 3). This was achieved by concurrently visualizing aligned 3D images and MRI data while the US probe performed vertical sweeps, progressively advancing from the reference point anteriorly (Fig. 2c). All image analyses were performed by two anatomists, each possessing over five years of experience. To assess the reproducibility of our methodology, the intra- and interobserver reliabilities were evaluated. Two independent evaluators analyzed the US images and repeated their assessments after a two-week interval. The intraclass correlation coefficient (ICC) was calculated to determine both intra- and inter-observer reliability. The ICC was computed using a two-way random-effects model (ICC[2,1]), and the value was 0.95 with a 95% confidence interval of 0.91–0.98. A supplementary video demonstrates the real-time tracking environment, showing the simultaneous acquisition of US and motion data using a marker-based motion capture system and tracked intraoral transducer.

## Results

**Fig. 4:**
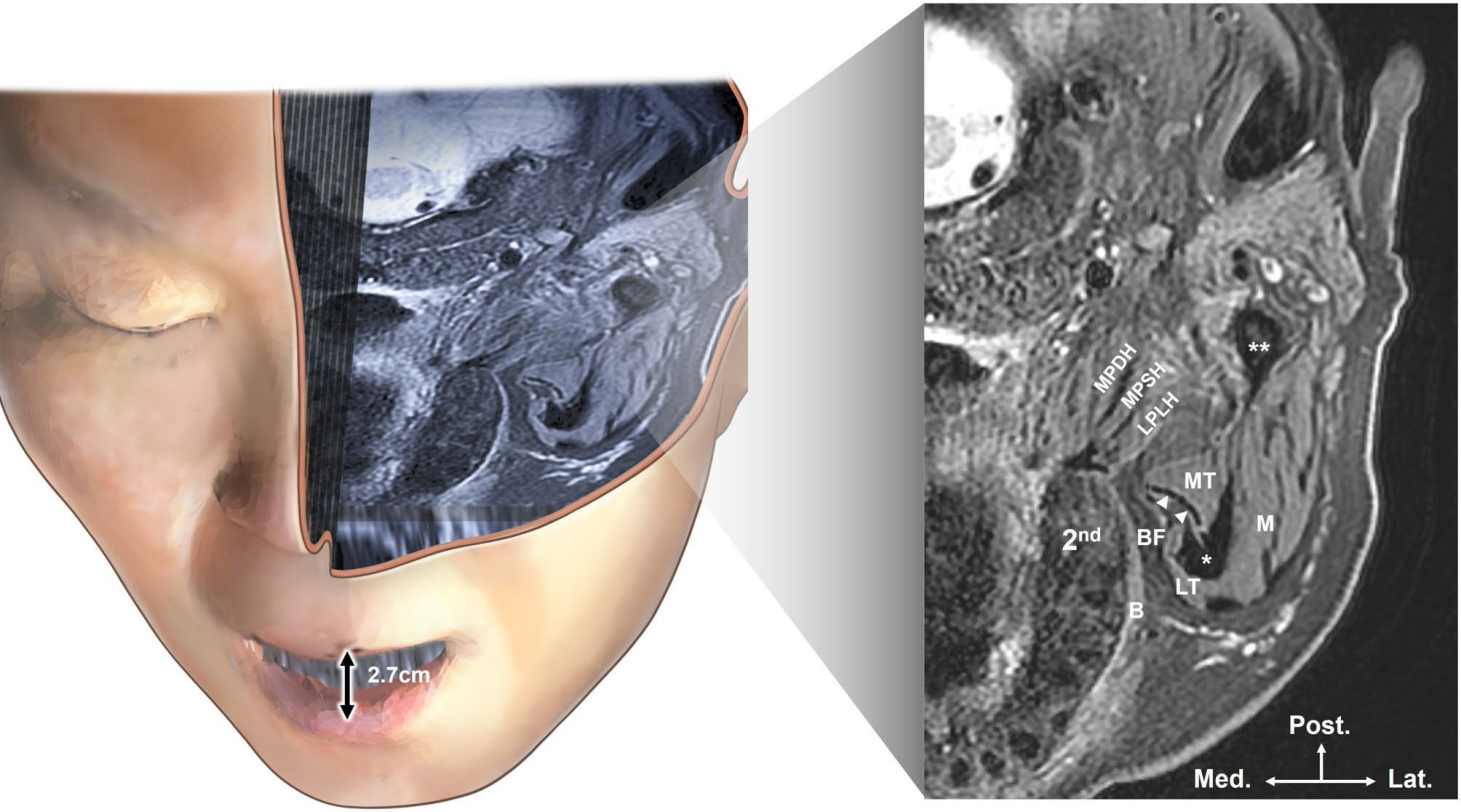
Superimposed model of the three-dimensional scanned face and magnetic resonance imaging (MRI), and MRI structures observed at the reference point level. second, maxillary second molars; MPDH, deep head of medial pterygoid muscle; MPSH, superficial head of medial pterygoid muscle; LPLH, lower head of lateral pterygoid muscle; B, buccinator muscle; BF, buccal fat pad; MT, medial portion of temporalis muscle; LT, lateral portion of temporalis muscle; M, masseter muscle; Asterisk, coronoid process; Two asterisk, condylar process; arrowhead, temporalis tendon; Med, medial; Post, posterior; Lat, lateral.

Anatomical structures were observed (Fig. 4) after superimposing the 3D scanned facial image with the MRI and US images at the reference point level. Figure 4 illustrates the integration of 3D surface data, MRI, and US images at the reference point, enabling clear anatomical visualization of the temporalis muscle, tendon, and surrounding bony structures. A supplementary video provides information on the superimposition of the three images.

### The 3D surface model was superimposed onto the MRI

Muscles exhibited high intensity, whereas teeth, tendons, and bones showed low intensity. Both medial and lateral portions of the temporalis muscle were identified on the lateral side of the maxillary second molar, with the temporalis tendon displaying low intensity between them. Additionally, the coronoid process and the posteriorly situated condylar process exhibited low intensity. The masseter muscle is located laterally between these two processes. Finally, on the distal side of the maxillary second molar, the lower head of the lateral pterygoid muscle, along with the deep and superficial heads of the medial pterygoid muscle, exhibited high intensity.

### US image with confirmed sSpatial information

**Fig. 5:**
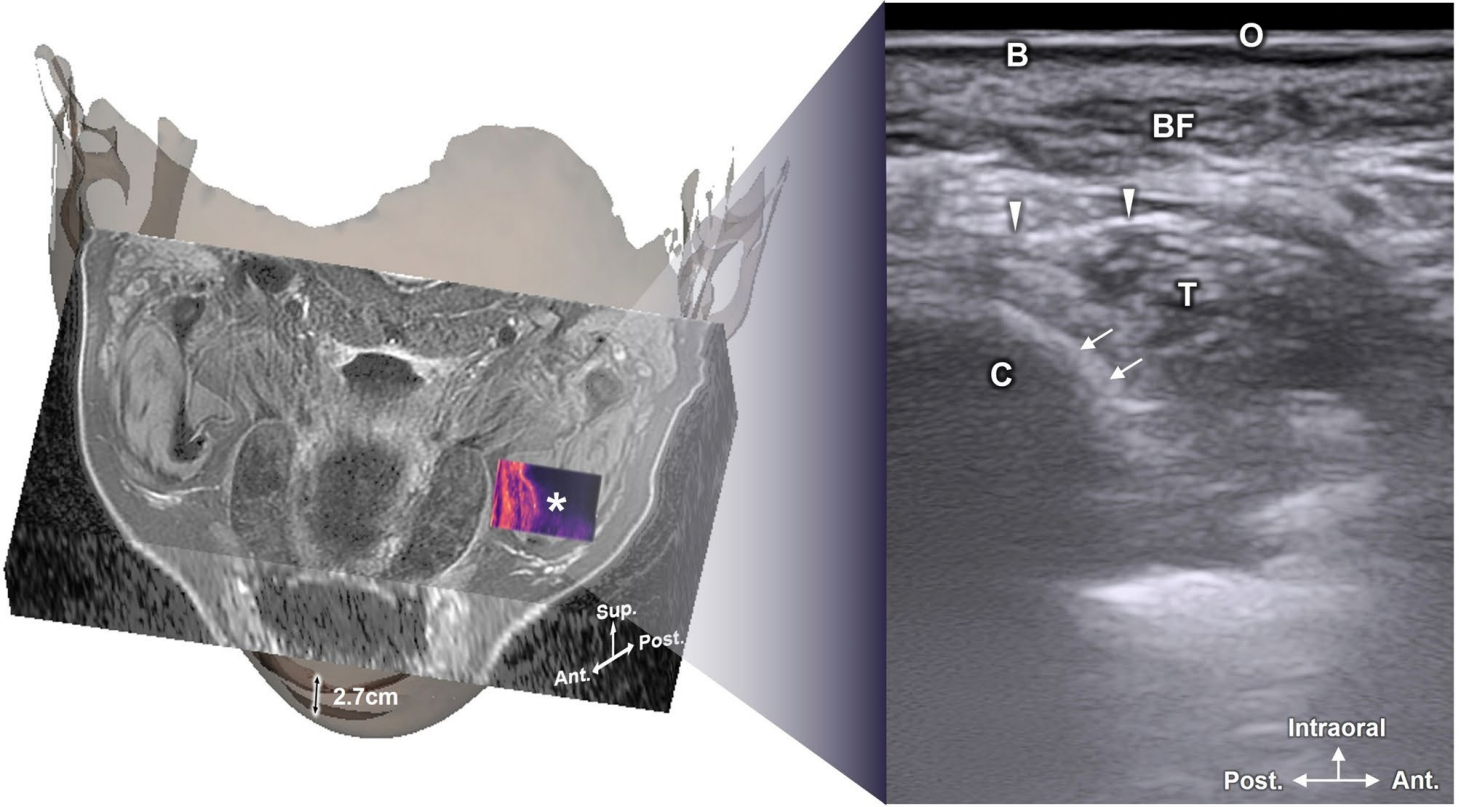
Superimposed model of the magnetic resonance imaging and a three-dimensional scanned face with tracked intraoral ultrasonography image. Ant, anterior; Post, posterior; Sup, superior; Asterisk, tracked US image; C, coronoid process; T, temporalis muscle; B, buccinator muscle; BF, buccal fat pad; O, oral mucosa; arrow, anterior border of coronoid process; arrowhead, temporalis tendon.

Figure 5 shows representative intraoral US images acquired at the LC and RC points, highlighting the coronoid process and surrounding soft tissue structures with confirmed spatial localization. The surrounding complex anatomical structures of the coronoid process were identifiable on all US images at the LC and RC points. The oral mucosa and buccinator muscle were visualized in the superficial plane, with the mucosa appearing hyperechoic and the buccinator muscle hypoechoic. The coronoid process manifested as a prominently hyperechoic structure, with the hypoechoic temporalis muscle situated directly above it. Hyperechoic temporalis tendons were also identified within the muscle. The buccal fat pad exhibited irregular hyperechoic patterns above the temporalis muscle (Fig. 5).

To assess the reliability of US image analysis, both intra- and inter-observer reliabilities were assessed. The ICC for intra- and inter-observer reliabilities was 0.95, demonstrating excellent agreement in US image interpretation. These results demonstrate the high reproducibility of our approach and reinforce the reliability of motion capture-assisted intraoral US imaging.

## Discussion

Given the highlighted advantages of US examinations, numerous efforts have been made to implement US within the oral cavity in the dental field^3–5,9,10^. Despite increasing recognition of its diagnostic and therapeutic value in the oral and maxillofacial regions, US devices are still underutilized in current dental clinical practice. The main barrier is the absence of standardized criteria for analyzing intraoral US images, leading to inconsistencies among researchers and challenges in image interpretation. US images exhibit considerable variability in quality, depending on the operator’s skill and experience, rendering their interpretation highly operator-dependent and requiring significant expertise.

Acquiring intraoral US images requires maintaining an open mouth and adjusting the position of adjacent structures, particularly those related to the temporomandibular joint. These positional adjustments complicate the accurate identification of anatomical features, underscoring the critical importance of precise localization data. Recent progress in 3D imaging and motion capture technologies has markedly enhanced the spatial accuracy and visualization of anatomical structures, facilitating precise localization for both research and clinical purposes^11–13^.

In this investigation, as a proof-of-concept feasibility study, we first developed a reference 3D model to validate the structures derived from 2D intraoral US images. This validation involved performing MRI scans with the mouth open to match the dimensions used during US imaging. Additionally, a 3D scanner was utilized to capture the facial morphology of the participant, and motion capture cameras tracked the positions of the US probe. These data modalities were overlaid to allow detailed comparative analyses. The outcomes enabled the detection of hypoechoic structures that were previously difficult to discern, such as the temporalis tendon. Moreover, US images obtained at the level of the maxillary second molar confirmed the absence of visibility for the medial and lateral pterygoid muscles (Figs. 4 and 5).

Motion capture technology, in particular, improves anatomical identification compared to traditional intraoral US by providing precise, real-time spatial localization of the US probe. This approach tracks the precise position and orientation of the US probe relative to the anatomical landmarks, which enables clinicians and researchers to accurately correlate the 2D US images with the corresponding anatomical structures. This spatial precision minimizes variability and facilitates the accurate and reproducible identification of complex intraoral anatomical structures that are challenging to distinguish with conventional US alone. Additionally, these precise positional data can be seamlessly integrated with other 3D imaging modalities (e.g., MRI and surface scanning), further enhancing anatomical clarity and reliability.

Currently, intraoral US research is limited, creating a gap in standardized imaging protocols and educational resources. The integration of motion capture technology proposed in this study effectively bridges this gap, facilitating precise anatomical localization and enhancing the real-time interpretation of intraoral US images.

Clinically, this technique exhibits significant potential for improving procedural accuracy in temporomandibular joint evaluations, periodontal assessments, implant site planning, and minimally invasive procedures such as US-guided injections. Moreover, by providing non-invasive, radiation-free, real-time visualization of soft tissue structures, this approach holds substantial promise for both clinical practice and anatomical education, benefiting dental clinicians and educators alike.

The captured data can also be stacked into 3D models (Fig. 2c). However, the accuracy of 3D US-derived models remains inferior to that of CT or MRI, primarily due to prolonged scanning times and tissue distortion. Extended scanning durations can introduce artifacts from patient movements, including breathing, pulsation, or involuntary muscle contractions. Additionally, subtle shifts in the position of the probe can distort soft tissue images. To reduce image artifacts and improve anatomical clarity, both real-time error correction techniques—such as motion compensation systems and predictive tracking models—and post-processing strategies using deep learning-based segmentation and enhancement algorithms should be considered^14^. These complementary approaches can minimize soft tissue distortion during acquisition and enhance the spatial accuracy of intraoral US imaging. Implementing intraoral stabilization devices, including probe holders or adjustable bite blocks, can further improve the consistency and reliability of intraoral 3D US imaging^14,15^.

The study was limited by a small sample size (*n* = 2) and the exclusive inclusion of healthy adults, which restricts the generalizability and clinical applicability of the findings to pathological conditions such as temporomandibular disorders. Future research should include proper sample size calculations and recruit participants with diverse anatomical and pathological characteristics to validate the broader clinical utility of this methodology. Nevertheless, the initial data suggest that this approach is promising for addressing the complexities of spatial relationships in intraoral imaging.

To advance intraoral US applications and address current limitations, future research should focus on the following key directions:

- Real-time error correction methods, such as motion compensation systems, are needed to reduce imaging artifacts caused by soft tissue motion and probe instability.
- Deep learning–based segmentation and enhancement algorithms could improve image clarity and reproducibility.
- Broader clinical trials with diverse patient populations will enhance the statistical validity and clinical relevance of future studies.

In conclusion, this study introduced an innovative anatomical analysis method that integrates motion capture technology with intraoral US and 3D imaging. By addressing current challenges in probe localization and spatial accuracy, this approach provides foundational data for standardizing intraoral US, which is a relatively underexplored domain.

## Electronic supplementary material

Below is the link to the electronic supplementary material.

Supplementary Material 1

Supplementary Material 2

## Data Availability

The data presented are available on request from the corresponding author.
