# Supplementary material for: Integrating motion capture technology and intraoral ultrasonography for 3D anatomical analysis
Source: Sci Rep. 2025 Jul 2;15:22813. doi: 10.1038/s41598-025-05763-x (PMC12217969; doi:10.1038/s41598-025-05763-x)
Supplement: Supplementary file 2 — Supplementary Material 2 [file 41598_2025_5763_MOESM2_ESM.docx]

**SUPPLEMENTARY VIDEO**

Supplement video 1. Real-time tracking of the intraoral ultrasonography probe using a marker-based motion capture system. The tracked probe movement is visualized in three-dimensional (3D) and superimposed onto magnetic resonance imaging and 3D facial scan images to demonstrate spatial alignment during intraoral scanning.
